# Unraveling the epigenomic and transcriptomic interplay during alcohol-induced anxiolysis

**DOI:** 10.1038/s41380-022-01732-2

**Published:** 2022-09-12

**Authors:** Harish R. Krishnan, Huaibo Zhang, Ying Chen, John Peyton Bohnsack, Annie W. Shieh, Handojo Kusumo, Jenny Drnevich, Chunyu Liu, Dennis R. Grayson, Mark Maienschein-Cline, Subhash C. Pandey

**Affiliations:** 1 Center for Alcohol Research in Epigenetics, Department of Psychiatry, University of Illinois at Chicago, Chicago, IL 60612 USA; 2 Jesse Brown Veterans Affairs Medical Center, Chicago, IL 60612 USA; 3 High-Performance Biological Computing, Roy J. Carver Biotechnology Center, University of Illinois-Urbana Champaign, Urbana, IL 61801 USA; 4 Research Informatics Core, University of Illinois at Chicago, Chicago, IL 60612 USA; 5 Present Address: Department of Psychiatry, SUNY Upstate Medical University, Syracuse, NY 13210 USA

**Keywords:** Neuroscience, Addiction

## Abstract

Positive effects of alcohol drinking such as anxiolysis and euphoria appear to be a crucial factor in the initiation and maintenance of alcohol use disorder (AUD). However, the mechanisms that lead from chromatin reorganization to transcriptomic changes after acute ethanol exposure remain unknown. Here, we used Assay for Transposase-Accessible Chromatin followed by high throughput sequencing (ATAC-seq) and RNA-seq to investigate epigenomic and transcriptomic changes that underlie anxiolytic effects of acute ethanol using an animal model. Analysis of ATAC-seq data revealed an overall open or permissive chromatin state that was associated with transcriptomic changes in the amygdala after acute ethanol exposure. We identified a candidate gene, *Hif3a* (Hypoxia-inducible factor 3, alpha subunit), that had ‘open’ chromatin regions (ATAC-seq peaks), associated with significantly increased active epigenetic histone acetylation marks and decreased DNA methylation at these regions. The mRNA levels of *Hif3a* were increased by acute ethanol exposure, but decreased in the amygdala during withdrawal after chronic ethanol exposure. Knockdown of *Hif3a* expression in the central nucleus of amygdala attenuated acute ethanol-induced increases in *Hif3a* mRNA levels and blocked anxiolysis in rats. These data indicate that chromatin accessibility and transcriptomic signatures in the amygdala after acute ethanol exposure underlie anxiolysis and possibly prime the chromatin for the development of AUD.

## Introduction

Clinical and preclinical studies have shown that anxiolytic effects of acute ethanol promote higher alcohol consumption and are crucial for the development of alcohol use disorder (AUD) [1–4]. Humans with anxiety disorders have the tendency to transition from occasional drinking to dependence more rapidly than those without [5, 6]. The extended amygdaloid circuitry is implicated in the regulation of anxiety behavior and serves as a critical hub in mediating acute and chronic ethanol’s behavioral and molecular effects [7–9]. Alcohol’s actions on modulating the excitability of the extended amygdaloid circuitry may play an important role in anxiety behaviors [10]. It is well established that the behavioral effects of acute and chronic alcohol exposure are under complex epigenetic control [2, 9].

We recently showed acute ethanol-induced anxiolysis is dependent on microRNA-494 (miR-494) signaling, which impacts regulation of CBP [CREB (cyclic adenosine monophosphate response element binding protein) binding protein], p300, and Cbp/p300-interacting transactivator 2 (CITED2) in the amygdala [11]. Acute ethanol exposure is associated with increases in histone H3K9 and H4K8 acetylation, via inhibition of histone deacetylases (HDACs), in addition to decreases in H3K9me2 and G9a (histone methyltransferase) protein levels in the amygdala of rats [12–15]. Recent studies have shown similar increases in histone acetylation in mouse hippocampus, prefrontal cortex and liver after acute ethanol exposure [16]. Furthermore, the increased histone acetylation in the amygdala and associated behavioral changes such as decreased anxiety undergo neuroadaptation (tolerance) and are reversed following withdrawal from chronic ethanol exposure [2, 12, 13]. However, in the amygdala, the genome-wide loci where these epigenomic and associated transcriptomic changes occur following a low dose of ethanol are currently unknown.

In this study, we sought to investigate this question by utilizing two genome-wide approaches: Assay for Transposase-Accessible Chromatin with high throughput sequencing (ATAC-seq), which identifies changes in genome accessibility, and RNA-seq, which assesses changes in global gene expression following acute ethanol [17, 18]. ATAC-seq makes use of the integrative capabilities of the hyperactive Tn5 transposase and next-generation sequencing to identify more accessible (open) versus less accessible (closed) regions across the genome and also provides a wealth of information regarding protein-DNA interactions [17, 19]. By functionally linking this with data from RNA-seq, which provides a global transcriptomic readout, we identified candidate gene targets containing differential chromatin modifications at their transcriptional control regions that correlated with differential expression in the amygdala, likely contributing to the anxiolytic effects of acute ethanol.

## Materials and methods

### Acute ethanol exposure paradigm

Adult male and female Sprague–Dawley rats were purchased from Harlan (Indianapolis, IN) and group housed with *ad libitum* food and water access in a temperature-controlled room on a 12/12-h light/dark cycle. All procedures were performed in accordance with the National Institute of Health Guidelines for the Care and Use of Laboratory Animals and approved by the Institutional Animal Care and Use Committee. Animals were randomly assigned to various groups and the number of rats in each group was based on our previous publications [11–13]. Acute ethanol (1 g/kg, intraperitoneal) or n-saline injections and anxiety behavioral measurements [elevated plus-maze (EPM) exploration test] were performed as described previously [13]. Rats were subjected to explore EPM for 5 min 1 h after injections. Immediately after behavioral measurements or 1 h after ethanol injections (without behavioral measurements), animals were anesthetized with isoflurane, and brain regions [amygdala (central, medial, and basolateral nuclei), bed nucleus of the stria terminalis (BNST) and dorsal hippocampus] were dissected (within 5 min) and stored at −80 °C until processed for various biochemical studies as described below. The Analox Alcohol Analyzer (Lunenburg, MA) was used to measure blood alcohol levels at the time of brain collection. The average blood ethanol level (mg%) of ethanol-treated male rats was 89.3 ± 2.9 (*n* = 40; mean ± SEM), as we have previously observed (11, 13–15). The average blood ethanol level (mg%) of ethanol-treated female rats was 74.5 ± 7.6 (*n* = 8; mean ± SEM), The mean ± SEM (*n* = 41) body weights (gm) of both treatment groups were not significantly different from each other in males (Control = 304.6 ± 4.3; Ethanol = 302.8 ± 3.6). Similarly, in females, the mean ± SEM (*n* = 8) body weights (gm) among both groups were not different from each other (Control = 204 ± 2.8; Ethanol = 200.6 ± 3.1). All data presented here were generated in adult male rats except Fig. S3, which was performed in adult female rats. Experiments were not performed blindly.

### ATAC-sequencing

Following acute ethanol treatment, frozen amygdala samples (six controls and six ethanol treated) were tagmented with Illumina’s Nextera DNA sample prep kit (Illumina®, San Diego, CA). Following purification, DNA was amplified using custom Nextera library-specific primers. The resulting libraries were assessed for quality nucleosome ladder profiles and quantification on the Agilent Bioanalyzer with the Agilent DNA HS assay kit (Agilent Technologies, Santa Clara, CA). Libraries were paired-end sequenced using the Illumina® platform. The bioinformatic processing of ATAC-seq data is described in supplementary methods.

### RNA-sequencing

Total RNA was extracted from amygdala tissues obtained from a separate cohort of rats, using the RNeasy® kit (QIAGEN, Germantown, MD) following the manufacturer’s protocol. A total of six controls (saline-treated) and six ethanol-treated rats were used. Agilent Tapestation (Agilent Technologies, Santa Clara, CA) was used to assess RNA quality using the RNA Integrity Number (RIN) assessment, and the average RIN value for the samples indicate high quality and integrity of isolated RNA (Control = 8.7 ± 0.118; Ethanol = 8.9 ± 0.119). Total RNA from 2 animals was pooled, resulting in *n* = 3 in each group for RNA-seq. The concentrations were determined by fluorometric analysis, and 1 µg of RNA in 20 µl of RNAse-Free water was used for library preparation. The libraries were prepared and sequenced in the DNA Services laboratory of the Roy J. Carver Biotechnology Center at the University of Illinois at Urbana-Champaign. The Illumina® platform was used for library preparation and single-end sequencing using the TruSeq Stranded mRNAseq Sample Prep kit (Illumina®, San Diego, CA). Libraries were sequenced for 100nt on three lanes of a HiSeq2500 (Illumina® platform). The bioinformatic analysis of RNA-seq data is described in supplementary methods.

### Integration of ATAC-seq with RNA-seq data

ATAC peaks were annotated to nearby genes on the basis of close proximity (promoter association, 2 kb ± from transcription start site [TSS]) or potential long-range interactions (distal association, up to 200 kb from TSS). The genes in the 2 kb list (31 genes, Table 1, FDR < 0.2) were cross-referenced for their presence in the RNA-seq data (FDR < 0.2) for mRNA changes.

**Table 1:** Genes that are associated with ATAC-seq peaks at their promoters (TSS ± 2 kb).

Peak ID	Chromosome	Start	End	Gene name	TSS ± 2 kb	Ethanol/Control: logFC	Ethanol/Control: *Q* Value
peak_3217	1	78998888	79004845	Hif3a	−1019	0.739398	1.51668E−10
peak_68740	20	8090968	8098770	Fkbp5	0	0.563998	1.33121E−05
peak_40629	15	30183087	30184570	LOC290156	0	0.613106	0.000106
peak_40797	15	31685828	31687526	LOC108353020	148	0.775034	0.000129
peak_108576	8	53142419	53148464	Zbtb16	0	0.419321	0.002019
peak_40843	15	32135389	32137992	LOC102556508	0	0.482184	0.002740
peak_40647	15	30553539	30555161	Trav14s1	677	0.779843	0.003097
peak_40834	15	32042566	32045051	LOC103693854	0	0.460100	0.005350
peak_40541	15	29366687	29370177	LOC684995	16	0.555889	0.006129
peak_63413	2	140705353	140708314	Mgst2	−82	0.536870	0.009204
peak_3216	1	78995896	78998702	Hif3a	0	0.431469	0.010197
peak_96228	6	76175484	76176788	Psma6	1024	0.605321	0.012949
peak_40796	15	31675907	31679085	LOC102555014	0	0.393897	0.014117
peak_10302	1	221753875	221755151	Pygm	−1174	0.685527	0.037892
peak_44676	16	8497159	8500489	Ogdhl	0	0.378384	0.073691
peak_67100	2	239413152	239414771	Cxxc4	−111	−0.417057	0.078753
peak_86677	5	33180626	33182976	Maged2	0	−0.317445	0.082602
peak_62601	2	105000505	105002148	LOC682402	0	−0.552217	0.100118
peak_105292	7	130540544	130541153	Acr	−167	0.764952	0.100712
peak_40773	15	31510679	31512130	LOC682708	0	0.482470	0.112276
peak_87750	5	69832439	69834831	Nipsnap3b	0	−0.451526	0.112958
peak_44673	16	8466095	8467877	LOC102550503	0	0.482126	0.123118
peak_9080	1	201498140	201500195	Htra1	0	0.371520	0.134251
peak_21401	11	30362069	30365302	Sod1	0	−0.284925	0.161426
peak_92245	5	165026989	165029576	LOC108351059	0	0.393443	0.165461
peak_97599	6	111475449	111477839	Slirp	0	−0.353192	0.165461
peak_97599	6	111475449	111477839	Alkbh1	0	−0.353192	0.165461
peak_40642	15	30533803	30535711	LOC102552674	0	0.512589	0.170042
peak_103519	7	109203060	109206986	Zfat	525	0.314836	0.181991
peak_1056	1	31493765	31495003	LOC100911237	0	−0.690268	0.185516
peak_34139	14	7617155	7618444	Slc10a6	0	0.602676	0.190299

### Candidate gene analysis for mRNA, histone acetylation, and DNA methylation fold changes

Total RNA was extracted, as described above, from the amygdala, BNST, and dorsal hippocampus. Reverse transcription followed by candidate gene qPCR to evaluate mRNA fold changes were performed, as described previously [11]. The occupancy of acetylated histones (H3K9/14ac, Cat. No. 06-599 MilliporeSigma, Burlington, MA; H3K27ac, Cat. No. 39133, Active Motif, Carlsbad, CA) and HIF3A (Cat. No. ab10134, Abcam, Waltham, MA) was evaluated at specific sites of genes using chromatin immunoprecipitation (ChIP) assay in the amygdala followed by qPCR and relative fold changes were determined, as described previously [20, 21]. DNA methylation fold changes were analyzed at the promoter sites of *Hif3a* and *Slc10a6* genes in the amygdala using MethylMiner™ assay for 5-methylcytosine (5mC) followed by qPCR and determination of relative fold changes, as published by us [22]. For all the qPCR assays, relative fold changes were determined using the ΔΔCt method [23]. Primer sequences are included in Table S4.

### Stereotaxic surgery and infusion of Hif3a siRNA into CeA

Animals were bilaterally cannulated with CMA/11 guide cannulae (CMA Microdialysis, North Chelmsford, MA) targeting the central nucleus of amygdala (CeA) using coordinates from bregma (posterior −2.5 mm, medial-lateral ±4.2 mm, ventral −5.1 mm) and siRNA infusion procedures as described previously by us [21, 24]. One week after surgery, they were infused with either negative control siRNA (Cat. No. 1027310) or Hif3a siRNA (Cat. No. SI01521604) (1 µg in 0.5 µl/side) (QIAGEN, Germantown, MD) using probes that delivered siRNA into the CeA, 3 mm beyond the guided cannulas [24]. The sequences of the Hif3a siRNA were: sense, 5′-CGACGAGAGGAUUGCAGAATT-3′; antisense 5′-UUCUGCAAUCCUCUCGUCGCA-3′. The siRNAs were dissolved in iFect solution (Neuromics, Edina, MN). Post-infusion (~23 h), rats were treated with ethanol (1 g/kg) or saline, and after 1 h, anxiety measures were determined using EPM [13]. Following behavioral measurements, rats were anesthetized with isoflurane, and amygdaloid tissues (predominantly CeA but also including surrounding medial and basolateral amygdala) were dissected and stored at −80 °C for mRNA measurements as described above.

### Chronic ethanol treatment

Adult male rats received liquid Lieber–DeCarli ethanol or control diets for 15 or 16 days as described previously [13, 14]. Ethanol-fed rats were withdrawn for 0 or 24 h, anesthetized, and sacrificed to dissect out the amygdala for the measurement of mRNA levels of *Hif3a* using qPCR, as described above.

### Statistical Analysis

Statistical analysis was performed using SigmaStat software suite (Systat Software Inc., San Jose, CA). Differences between control and ethanol groups were analyzed using Student’s unpaired two-tailed *t* test. For Hif3a siRNA experiments, two-way ANOVA followed by Tukey’s post-hoc test was performed. For the *Hif3a* chronic ethanol and withdrawal experiment, Kruskal–Wallis one-way analysis of variance followed by Tukey’s post-hoc test was used. Inclusion/exclusion criteria were not preestablished. Outliers were determined using interquartile range (IQR) method. For whole genome approaches (ATAC-seq and RNA-seq), details of statistical analysis are provided in supplementary methods.

## Results

### Acute ethanol produces an anxiolytic-like response

We measured anxiety-like behaviors 1 h after acute ethanol by using the elevated plus maze exploration (EPM) test. The results confirmed our earlier findings [11, 13–15] that acute ethanol significantly increases the percentage of open-arm entries and percentage of time spent in the open arms (Fig. S1), which are indicators of the anxiolytic effects of acute ethanol in male rats. Similar findings were also observed in female rats (Fig. S3A).

### ATAC-seq analysis in the amygdala after acute ethanol exposure

**Fig. 1:**
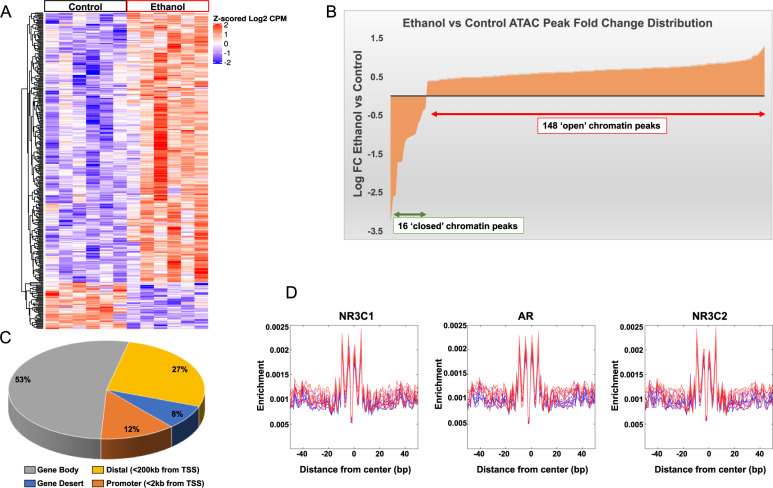
ATAC-seq data analysis in the amygdala of acute ethanol exposed rats. **A** Heatmap showing differential ATAC peaks (FDR < 0.2; 345 peaks) and chromatin accessibility in the amygdala after acute ethanol exposure. Red represents increases in peaks and blue represents decreases in peaks. Individual peaks are represented in rows and individual animals from control and ethanol groups (*n* = 6 animal per treatment) are shown in columns. **B** Distribution of ATAC peak fold changes indicating that out of the 164 peaks at FDR < 0.05, 148 peaks were associated with ‘open’ chromatin regions in the amygdala of acute ethanol-treated compared to 16 peaks in the control rats. **C** ATAC-seq data analysis in the amygdala of acute ethanol exposed rats showing distribution of ATAC peaks across genomic features. **D** Footprinting images for the top 3 (out of 41) motifs derived from FIMO analysis of 572 vertebrate motifs from the JASPAR database. The motifs satisfied the following criteria: *q* < 0.01 and >50% enrichment (log2 ratio >0.585). The blue lines represent controls and the red lines represent ethanol-treated samples. [(NR3C1- Glucocorticoid Receptor; Nuclear Receptor Subfamily 3 Group C Member 1), (AR- Androgen Receptor), NR3C2 (Mineralocorticoid Receptor; Nuclear Receptor Subfamily 3 Group C Member 2)].

We used ATAC-seq to assess chromatin accessibility in the amygdala after acute ethanol exposure and obtained a total of 118,836 peaks (Table S1). We observed a differential pattern of distribution (Heatmap, Fig. 1A) with an overall increase in genome-wide chromatin accessibility after acute ethanol exposure (Fig. 1A, B). The majority of peaks were localized to intergenic and distal regulatory regions, while about 12% of these peaks (14,707) were in promoter proximal regions (±2 kb of TSS) (Fig. 1C). We observed 164 significant peaks that were differentially altered in the amygdala between ethanol-treated and control rats at FDR < 0.05: 148 were ‘open’-chromatin peaks in ethanol-treated versus saline-treated controls and 16 were ‘closed’ (Fig. 1B). We identified 31 genes (FDR < 0.2) that had peaks at their promoters (±2 kb of TSS) (Table 1). Ingenuity Pathway Analysis (IPA®) of these genes resulted in two gene networks (Fig. S4) containing *Hif3a* (hypoxia-inducible factor 3 subunit alpha) and *Slc10a6* (solute carrier family 10 member 6). *Hif3a* promoter contained two peaks (peak_3217 and peak_3216) and *Slc10a6* contained one peak (peak_34139) that were upregulated after acute ethanol exposure (Table 1). Interestingly, the most significant differential promoter peak was associated with *Hif3a*, a transcription factor involved in adaptive responses to hypoxic stress impacting diverse regulatory biological pathways [25, 26]. These data suggest that expression of these two genes may be altered in the amygdala, most likely induced by acute ethanol exposure.

We further searched for putative transcription factor (TF) binding motifs in the differential ATAC peak regions (FDR < 0.2; 345 peaks) to identify potential regulators of differential chromatin accessibility in the amygdala, and we also generated footprinting images to supplement these data. True TF-DNA binding should prevent integration of the transposase at the binding site, which is reflected in these footprints as a drop-in enrichment or a pattern change around the motif that matches the size of the binding site. The observed reduction in transposon binding at the site of the motif then implies occupancy of those sites by another molecule, which, based on the motif specificity, is most likely the TF [17] (Fig. 1D and Table S2). Predicted transcription factors that bind these motifs include those that have been implicated in alcohol responses, such as the glucocorticoid receptor, NR3C1 [27–29].

### RNA-seq analysis and validations of gene expression in the amygdala after acute ethanol exposure

**Fig. 2:**
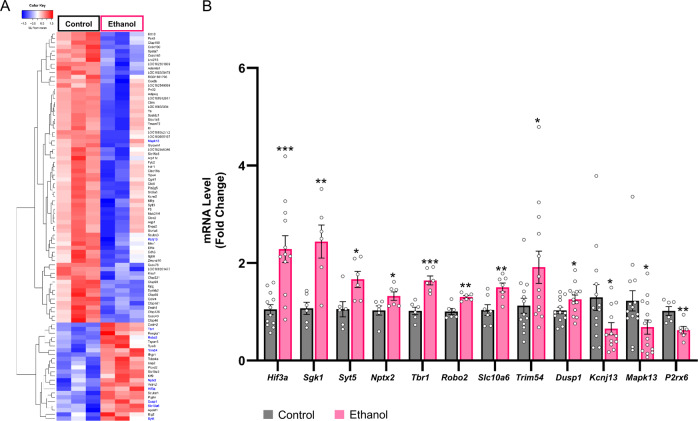
RNA-seq data analysis in the amygdala of acute ethanol-exposed rats. **A** HeatMap of RNA-seq differentially expressed transcripts at FDR < 0.2.Hierarchical clustering and heatmap of differentially expressed mRNAs (FDR < 0.2) from acute ethanol and normal saline (control) treated rats. Red represents increases in overall expression and blue represents decreases in expression. Individual mRNAs are represented in rows and individual experiments (*n* = 3 in each treatment; RNA from two rats are pooled for each treatment) are shown in columns. Gene names highlighted in blue were used for the validations. **B** Validations of mRNA levels of selected candidates from RNA-seq data in the amygdala of acute ethanol exposed rats. Expression levels of mRNAs were significantly different in the amygdala of acute ethanol-exposed rats compared to saline-treated control rats, as measured by qPCR. Data represents the mean ± SEM and individual values are shown on bar diagrams with circle dots for control and ethanol groups (*n* = 6–14; Two-tailed Student’s *t* test; *p < 0.05; **p < 0.01; ***p < 0.001).

To evaluate whether acute ethanol-induced chromatin accessibility is responsible for transcriptomic changes in the amygdala of male rats, we performed RNA-seq using the Illumina® platform. The high sequencing depth resulted in 62.58–70.76 million reads per sample (Fig. S5). After calculating fold changes and FDR corrected values (Table S3), we used an FDR < 0.2, to identify 91 genes that exhibited significant differential fold changes in the ethanol group compared to controls (Heatmap, Fig. 2A). Pathway analysis using IPA® revealed several networks involved in critical cellular processes including organismal injury, cell death and survival, cellular organization and tissue development. The top 4 networks are shown in Fig. S6. For mRNA validations, we selected candidate genes from the IPA® networks that had increased and decreased transcript levels after acute ethanol compared to controls: Increased—*Hif3a, Syt5* (Synaptotagmin 5)*, Tbr1* (T-box, Brain, 1)*, Trim54* (Tripartite Motif Containing 54)*, Robo2* (Roundabout Guidance Receptor 2)*, Nptx2* (Neuronal Pentraxin 2)*, Slc10a6* and *Dusp1* (Dual Specificity Phosphatase 1); Decreased—*Kcnj13* (Potassium Inwardly Rectifying Channel Subfamily J Member 13) and *Mapk13* (Mitogen-Activated Protein Kinase 13). Quantitative RT-PCR confirmed that the expression changes were similar to the changes seen in the RNA-seq data, thus validating the findings (Fig. 2B). We further validated two genes (FDR > 0.2), *Sgk1* (Serum/Glucocorticoid Regulated Kinase 1) and *P2rx6* (Purinergic Receptor P2X 6) (Fig. 2B) that exhibited increased and decreased expression, respectively, in ethanol-treated rats compared to controls. *Sgk1* is a known glucocorticoid response gene, and since our ATAC peak motif analysis identified putative NR3C1 (glucocorticoid receptor) binding motifs to be enriched, we chose this gene. Furthermore, *Sgk1* functions as an important modulator of ion channel function and neuronal plasticity and plays a role in regulating acute and chronic ethanol phenotypes via its actions in mouse prefrontal cortex [27]. In addition, we measured *Hif3a* mRNA in the amygdala of female rats and found that acute ethanol exposure significantly increased *Hif3a* mRNA levels (Fig. S3B), similar to male rats (Fig. 2B).

### Integration of ATAC-seq and RNA-seq analysis and validations

**Fig. 3:**
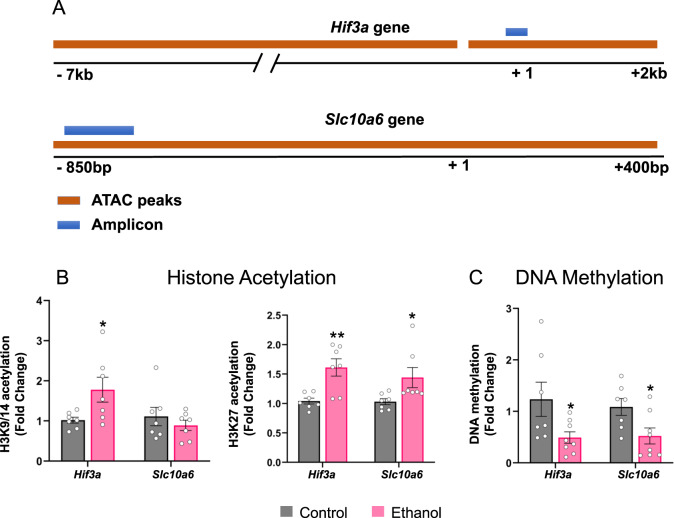
Histone acetylation (H3K9/14ac; H3K27ac) and DNA methylation (5-methylcytosine, 5mC) changes reveal relaxed chromatin architecture at the ATAC-seq peak regions near the transcription start site (TSS) of *Hif3a* and *Slc10a6* genes. **A** Schematic showing the location of ATAC-seq peaks and location of primers for qPCR validation for *Hif3a* and *Slc10a6*. **B** The “open peak” regions at the transcriptional control regions of *Hif3a* and *Slc10a6* were evaluated by chromatin immunoprecipitation (ChIP) assay representing fold changes of H3K27ac and H3K9/14ac occupancy at selected regions of *Hif3a* and *Slc10a6* in ethanol-treated and control rats. **C** Changes in DNA methylation were also evaluated at the same loci of *Hif3a* and *Slc10a6* in amygdala of ethanol-treated and control rats and represented as fold changes in DNA methylation (5mC levels). Values are represented as the mean ± SEM and individual values are shown on bar diagrams with circle dots for control and ethanol groups (*n* = 7–8; two-tailed Student’s *t* test *p < 0.05; **p < 0.01).

Amongst the 31 genes with differential chromatin peaks in their promoters (Table 1), two candidates that emerged from IPA, *Hif3a and Slc10a6*, also exhibited increased mRNA expression following acute ethanol compared to controls in both the RNA-seq data (FDR < 0.2) and qPCR validations (Fig. 2B). We used ChIP and DNA methylation assays to investigate the open-chromatin peak regions at the promoters of *Hif3a* and *Slc10a6* by designing primers within the peak locations for both *Hif3a* (peak that overlaps TSS) and *Slc10a6* (Table 1; Fig. 3A). For ChIP, we used antibodies against two well-characterized activating histone modifications, H3K9/14ac and H3K27ac [2, 9]. We observed an increase in H3K27ac occupancy at both *Hif3a* and *Slc10a6* (Fig. 3B) and an increase in H3K9/14ac occupancy at the *Hif3a* site, but no change in H3K9/14ac occupancy at the *Slc10a6* site (Fig. 3B). In addition, we observed a decrease in 5-methylcytosine (5mC) levels (Fig. 3C) at these locations of *Hif3a* and *Slc10a6*. These findings are suggestive of an “open” or permissive chromatin structure, as reflected by ATAC-seq data. These data suggest that increased histone acetylation and decreased DNA methylation at *Hif3a* and *Slc10a6* promoters in the amygdala may be associated with their observed increased mRNA levels after acute ethanol exposure.

### *Hif3a* gene knockdown in the CeA promotes anxiety and attenuates anxiolytic effects of acute ethanol

**Fig. 4:**
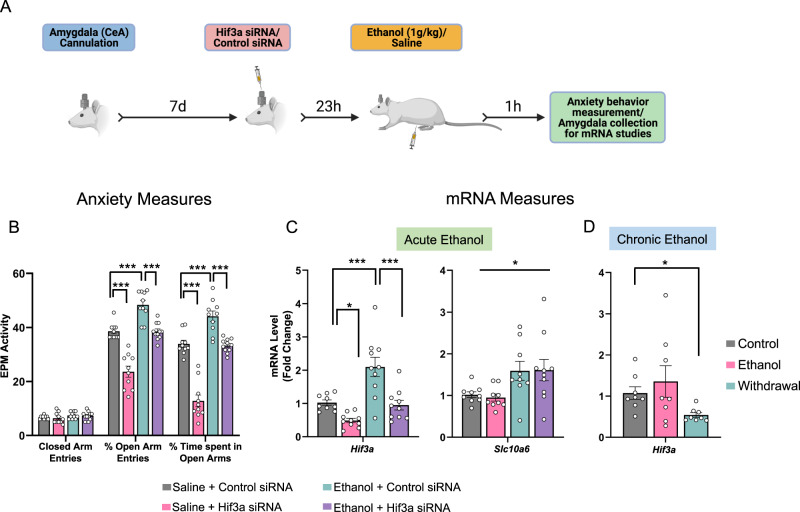
Hif3a siRNA infusion into the central nucleus of amygdala (CeA) blocks ethanol-induced anxiolysis and reduces mRNA levels of *Hif3a*, but not *Slc10a6*. *Hif3a* mRNA is decreased during withdrawal after chronic ethanol exposure. **A** Schematic representation of the experimental design for the siRNA infusion and behavioral measurement. **B** Bar diagram showing anxiety-like behaviors following Hif3a siRNA infusion into the CeA with or without acute ethanol treatment (1 g/kg, intraperitoneal) in rats. Hif3a siRNA infusion into CeA (24 h prior) prevented anxiolytic effects of acute ethanol in rats. Values are represented as mean ± SEM (*n* = 10–11; two-way analysis of variance (Percent open arm entries: treatment effect, *F*_1,37_ = 72.397, *p* < 0.001, group effect *F*_1,37_ = 71.445, *p* < 0.001, treatment × group interaction, *F*_1,37_ = 3.078, *p* = 0.088; Percent time spent in open arms: treatment effect, *F*_1,37_ = 97.836, *p* < 0.001, group effect, *F*_1,37_ = 90.159, *p* < 0.001, treatment × group interaction, *F*_1,37_ = 10.175, *p* < 0.01) followed by post-hoc Tukey’s test: ***p < 0.001). **C** The mRNA levels of *Hif3a* and *Slc10a6* were also measured in the amygdala of rats infused with control siRNA or Hif3a siRNA into CeA then treated with either saline or ethanol. Values are represented as mean ± SEM [*n* = 9–10; Two-way analysis of variance (*Hif3a*: treatment effect, *F*_1,35_ = 23.54, *p* < 0.001; group effect, *F*_1,35_ = 19.366, *p* < 0.001; *Slc10a6*: group effect, *F*_1,33_ = 11.003, *p* < 0.01) followed by post-hoc Tukey’s test: *p < 0.05; ***p < 0.001]. **D** Bar diagram showing changes in *Hif3a* mRNA levels in the amygdala of control, chronic ethanol-treated and ethanol-withdrawn (24 h) rats. Values are represented as mean ± SEM (*n* = 8; Kruskal–Wallis one-way analysis of variance on ranks (H_2_ = 7.3, *p* < 0.05) followed by Tukey’s test, *p < 0.05). Individual values are shown on bar diagrams with circle dots for various groups.

Results from merging the two big data sets suggest that ‘open’ ATAC-seq peaks (chromatin accessibility) may be responsible for increased mRNA levels of *Hif3a* in the amygdala after acute ethanol exposure. In addition, we observed that *Hif3a* mRNA levels were increased in BNST and hippocampal brain regions after acute ethanol exposure (Fig S2). We investigated whether induction of *Hif3a* mRNA in the adult male CeA is involved in the ethanol-induced anxiolysis (Fig. 4A), as this brain region is implicated in anxiety and alcohol-related behaviors [2, 8, 30]. It was found that Hif3a small interfering RNA (siRNA) infusion into the CeA significantly attenuated ethanol-induced increases in *Hif3a* mRNA levels and associated anxiolysis in rats (Fig. 4B, C). Interestingly, Hif3a siRNA infusion in ethanol-naïve control rats decreased *Hif3a* mRNA levels in the amygdala and provoked anxiety-like behaviors. The differential reduction in anxiety by Hif3a siRNA in control and alcohol-treated animals may be related to differential baseline anxiety levels. In order to ascertain whether these changes were specific to *Hif3a* mRNA expression, we also measured mRNA levels of *Slc10a6* in these rats and observed no changes in mRNA levels of *Slc10a6* in Hif3a siRNA infused rats (Fig. 4C). Taken together, these results suggest that increased expression of *Hif3a* in the CeA mediates acute ethanol-induced anxiolysis.

### Chronic ethanol exposure and withdrawal decrease *Hif3a* expression in the amygdala

We used an established animal model of alcohol dependence, wherein rats exhibit anxiety-like behaviors during withdrawal following chronic ethanol exposure, and measured *Hif3a* expression in these amygdala tissues [13, 14]. It was found that *Hif3a* mRNA levels were significantly decreased during withdrawal (24 h) compared to control animals, but that they were unchanged when ethanol was still in the system (0 h withdrawal; Fig. 4D). These data suggest that decreased *Hif3a* expression in the amygdala may be involved in development of ethanol withdrawal-related anxiety-like behaviors. This is mechanistically supported by the fact that infusion of Hif3a siRNA into the CeA provoked anxiety-like behaviors in alcohol naïve male control rats (Fig. 4B, C).

### Acute ethanol-induced HIF3A binding to *Npy1r* promoter induces *Npy1r* mRNA levels

Next we examined the molecular mechanisms by which HIF3A might be involved in the regulation of acute ethanol-induced anxiolysis. Recently, a HIF3A ChIP-seq study in human cell lines identified genome-wide HIF3A binding sites [31]. Based on this observation, we utilized the JASPAR database [32] to identify putative hypoxia response element (HRE) sites at the rat neuropeptide Y receptor Y1 (*Npy1r)* gene promoter (Fig. S7A). It has been shown that activation of *Npy1r* in the amygdala, particularly in the CeA, produces anxiolytic effects [33, 34]. Using an antibody against HIF3A, we performed the ChIP assay and observed increased HIF3A binding following acute ethanol in the vicinity of a predicted HRE site at the *Npy1r* promoter region in the amygdala (Fig. S7A, B). Expression levels for this gene trended to increase after acute ethanol exposure in RNA-seq data (FDR < 0.475; Table S3). We confirmed these results by measuring *Npy1r* mRNA levels following acute ethanol exposure and observed a significant increase in the amygdala (Fig. S7C). These data suggest the possibility that a HIF3A mediated increase in *Npy1r* mRNA levels in the amygdala could be involved in alcohol-induced anxiolysis.

## Discussion

**Fig. 5:**
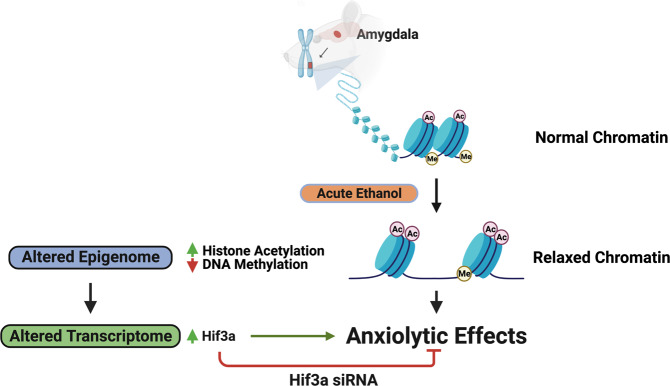
This model depicts the ability of acute ethanol to rapidly alter the epigenome in the amygdala and produce transcriptomic changes. These genome-wide chromatin accessibility and transcriptomic signatures in the amygdala are associated with anti-anxiety effects of ethanol in rats. One such candidate gene is hypoxia-induced gene transcription factor, *Hif3a*, which is epigenetically induced by acute ethanol, and anxiolytic effects associated with acute ethanol are prevented by inhibiting *Hif3a* expression in the central nucleus of amygdala of rats. The rapid and dynamic epigenomic modifications from a low dose of ethanol clearly suggest that these molecular processes may prime the amygdala to the emotional negative consequences that are most commonly associated with and promote alcohol use disorder.

The present study has identified epigenomic and transcriptomic changes in the amygdala produced by acute ethanol exposure as well as a candidate gene, *Hif3a*, that is mechanistically linked to the anxiolytic effects of ethanol. Here, we used next-generation sequencing approaches, ATAC-seq and RNA-seq, to demonstrate that acute ethanol has the ability to induce a predominantly ‘open’ chromatin structure in the amygdala, thereby facilitating DNA-protein interactions and altering the transcriptome (Fig. 5). Our earlier findings indicate that acute ethanol decreases HDAC activity and increases CBP levels with associated increases in H3K9ac and H4K8ac levels in rat amygdala [13, 15]. Corroborating these findings, our ATAC-seq analysis revealed an overall “open” or permissive chromatin state, in the amygdala of acute ethanol-exposed rats. Footprinting analysis further identified important ethanol-regulated transcription factor motifs such as the glucocorticoid receptor, NR3C1 [27–29]. It is known that TF motifs occur in intergenic regions, which interact with specific core promoter elements via chromatin looping [35], as revealed by approaches such as chromosome conformation capture (3C) and its derivatives [36]. RNA-seq revealed several candidate genes involved in critical cellular functions such as mediation of stress responses (*Hif3a*, *Sgk1*), synaptic signaling and communication (*Syt5*, *Nptx2*), and regulation of intracellular MAP kinase signaling (*Dusp1*).

Comparing ATAC-seq with RNA-seq data revealed two promising candidates, *Hif3a* (Hypoxia-inducible factor 3, alpha subunit) and *Slc10a6* (solute carrier family 10 member 6), which had increased chromatin accessibility around their TSS’ along with up-regulation of both transcripts after acute ethanol exposure in the amygdala. The ‘open’ chromatin regions at these promoters had differential histone modifications associated with them. Combinatorial histone modifications are typically loci specific [37], and here we observed that acute ethanol increased H3K27ac and H3K9/14ac levels at the promoter of *Hif3a* and H3K27ac but not H3K9/14ac at the promoter of *Slc10a6*. DNA methylation (5mC) was decreased at both gene promoters. SLC10A6 also known as sodium-dependent organic anion transporter (SOAT), is part of the SLC family of transporters, which are involved in active and passive transport of various inorganic ions, neurotransmitters, amino acids and various other substrates, and plays a role in the transport of sulfate steroids and associated signaling pathways [38]. Interestingly, other members of this family, such as the glutamate transporter *Slc17a7* and the GABA transporter *Slc6a11*, have been implicated in mediating alcohol responses [39, 40]. HIF3A is a transcription factor that is involved in adaptive responses to hypoxic stress and impacts multiple regulatory pathways [26]. Other members of this family, such as HIF1A, are known to interact with CITED2 (cAMP-responsive element-binding protein [CBP]/p300-interacting transcriptional modulator), which has been linked to the anxiolytic response to acute ethanol [11, 41]. Hypoxia response element (HIF binding) or HRE sites have been shown to occur in conjunction with cAMP response element-binding (CREB) sites, activator protein 1 (AP1), CCAAT-enhancer binding protein (CEBP) and ATF3 sites and TF’s binding these sites are thought to functionally interact and co-operate to affect hypoxia-induced changes in gene expression [41–43]. CREB signaling, as has been shown in various studies, is crucial for anxiety and alcohol comorbid phenotypes in rats via chromatin remodeling mechanisms [2, 9]. Here, we observed that *Hif3a* expression is increased in the amygdala during acute ethanol but is normalized after chronic ethanol exposure and significantly decreased during withdrawal. Similarly, we have previously reported that in the amygdala, acute ethanol exposure increased histone acetylation, CBP levels, and inhibited HDAC activity, which is normalized after chronic ethanol exposure. During withdrawal after chronic ethanol exposure, however, HDAC activity is increased and CBP levels are decreased, and histone acetylation also is decreased [11, 13, 15]. These data suggest that Hif3a as well as other transcriptomic changes that occur during acute ethanol exposure may undergo neuroadaptive processes [8] most likely involving dynamic epigenetic changes [2, 11, 13] after chronic ethanol exposure and subsequent withdrawal.

Inhibition of *Hif3a* expression in the CeA was able to attenuate acute ethanol-induced anxiolysis. A recent study [31] revealed HIF3A occupancy at transcriptional control regions of critical genes implicated in regulating alcohol and anxiety-related comorbid behaviors, such as neuropeptide Y (*Npy*) and activity-regulated cytoskeletal associated protein (*Arc*). Acute ethanol exposure has been shown to increase expression of *Npy* and *Arc* in the amygdala that is possibly involved in the anxiolytic actions of alcohol [13, 14]. The receptor for *Npy* is the *Npy1r* gene, and activation of NPY receptor Y1 with a NPY1R agonist produces anxiolysis [33, 34]. Furthermore, it has been shown that NPY via NPY1R activation suppresses the corticotropin-releasing factor (CRF) and modulates GABAergic pathways in the CeA to regulate anxiety and alcohol-related behaviors [8, 34, 44, 45]. We identified HRE sites at *Npy1r* control regions and tested this relationship. We observed increased HIF3A binding at the *Npy1r* promoter following acute ethanol exposure. Furthermore, this was associated with increased expression of *Npy1r* in the amygdala. These data suggest that HIF3A may regulate alcohol-induced anxiolysis via activating NPY1R signaling in the amygdala.

Data collected here suggest that inhibition of *Hif3a* expression in the CeA is sufficient to attenuate ethanol-induced anxiolysis, but involvement of other brain circuitry cannot be ruled out, as we observed that acute ethanol exposure increased *Hif3a* expression in BNST and hippocampus. These brain circuitries also play an important role in alcohol-related anxiety and emotional behaviors [8, 46, 47]. It is well established that there are sex differences in acute ethanol sensitivity [48–50], so we tested this idea and similar to male rats, acute ethanol exposure produced anxiolytic effects and increased *Hif3a* expression in the amygdala of female rats. These findings suggest that HIF3A may be a common target in mediating acute ethanol-induced anxiolysis in both sexes.

In summary, the combined use of two powerful genome-wide approaches, ATAC-seq and RNA-seq identified genomic loci that may be involved in the rapid epigenetic regulation of the transcriptome in the amygdala after acute ethanol exposure, suggesting that even a low dose of ethanol may impact the epigenome and drive processes responsible for addictive behaviors (Fig. 5). Clinically, it has been shown that acute alcohol priming causes high-risk social drinkers to demonstrate binge consumption behavior similar to heavy drinkers [51]. We identified an epigenetic target, *Hif3a*, in the amygdala that regulates anxiety-related behaviors after ethanol exposure. *Hif3a* is induced following acute ethanol, which is associated with anxiolytic-like behavior [11], and decreased during withdrawal after chronic ethanol exposure, which is associated with increased anxiety-like behavior [13, 14]. Interestingly, knockdown of *Hif3a* in the CeA induced anxiety-like behaviors in ethanol-naïve control rats, mimicking the ethanol withdrawal effects on these measures. These data provide the important link between *Hif3a* and ethanol-related anxiety behaviors (Fig. 5). This study also reiterates the fact that acute ethanol-induced epigenomic changes can serve as a molecular indicator of genes that could potentially become dysregulated upon chronic exposure and subsequent withdrawal. AUD comorbid with anxiety has a complex multifactorial etiology, and these data make a compelling argument to target HIF3A signaling in the amygdala for the development of pharmacological interventions in the treatment of AUD.

## Supplementary information

Supplemental Material

Table S1

Table S3

## Data Availability

All the data on which conclusions are based are included either in the main manuscript or in supplemental materials. The ATAC-seq (accession number GSE 171399) and RNA-seq (accession number GSE 171400) data sets have been deposited at gene expression omnibus (GEO) of NCBI (SuperSeries GSE accession number 171412).
