# Supplementary material for: Unraveling the epigenomic and transcriptomic interplay during alcohol-induced anxiolysis
Source: Mol Psychiatry. 2022 Sep 12;27(11):4624–32. doi: 10.1038/s41380-022-01732-2 (PMC9734037; doi:10.1038/s41380-022-01732-2)
Supplement: Supplementary file 1 — Supplemental Material [file 41380_2022_1732_MOESM1_ESM.pdf]

Supplementary Materials

Unraveling the epigenomic and transcriptomic interplay during alcohol-induced anxiolysis

Harish R. Krishnan, Huaibo Zhang, Ying Chen, John Peyton Bohnsack, Annie W. Shieh, Handojo Kusumo, Jenny Drnevich, Chunyu Liu, Dennis R. Grayson, Mark Maienschein-Cline, Subhash C. Pandey

Supplementary Methods:

Bioinformatics of ATAC-seq data

Reads were aligned to the Rattus norvegicus Rnor 6.0 genome using BWA-MEM (Burrows-Wheeler Aligner) with default parameters [1]. Read alignments were adjusted to account for transposon binding: + strand alignments by +4bp, - strand alignments by -5bp [2] and PCR duplicates were removed using Picard MarkDuplicates [3]. Peak calling was performed using Macs2 with parameters "--nomodel --nolambda" as appropriate for ATAC-seq data: no shifting model applied, and no input control sample [4]. Peaks were filtered at a -log10 q-value threshold >5. Peak calls from each replicate across both control and ethanol treatments were merged into a union set using bedtools merge [5]. This unified set of peaks was used as final genomic features for differential statistics and other downstream analyses. The unified peaks were annotated to genes from rn6 to provide the genic context for each peak, including promoter (+/-2kb from TSS), overlap with gene body (between TSS and TSE), upstream (<200kb upstream of TSS), or downstream (<200kb downstream of TSS). Peak abundance for each individual sample was quantified for the unified peak set as raw counts based on PCR duplicate-removed read alignments using featureCounts [6]. Normalization was performed to account for differences in sequencing depth across libraries, and reads were expressed as CPM (counts per million) with TMM normalization from edgeR [7]. Differential statistics were computed with edgeR using exactTest [7]. We adjusted p-values for multiple testing using the false discovery rate (FDR) correction [8].

Motif and Footprinting analysis of ATAC-seq peaks

We extracted genomic sequences from the unified peaks using samtools fax [9], and performed a search of all transcription factor (TF) motifs from the JASPAR core vertebrate motif database within those sequences using FIMO [10,11]. To identify putative transcription factors (TF) of interest within the ATAC peak regions, we computed enrichment statistics for each motif: we compared the fraction of differentially enriched peaks (FDR < 0.2, 345 peaks out of ~118k) containing a motif to the fraction of non-differentially enriched peaks containing the same motif, computing log2-odds ratios and p-values using Fisher's Exact test, and adjusting p-values using the FDR correction over all motifs tested. Positive enrichment ratios indicate that motifs are present within the differentially enriched peaks more frequently than expected by chance (a log2 ratio of 1 indicates that a motif occurs twice as frequently [2^1] as expected by chance) and corresponding FDR-corrected p-values measure the statistical significance of those

observations. To strengthen our motif enrichment analysis by providing additional evidence of TF binding to open chromatin regions in aggregate, we compiled an averaged genomic footprint across all motif hits from ATAC-seq read start positions for each base surrounding the motif and generated footprinting figures (Fig.1D) for the motifs with q<0.01 and >50% enrichment (log2 ratio > 0.585) (Table S2).

Bioinformatics of RNA-seq data

Quality control and trimming of the reads were done using FastQC (v0.11.8) using default parameters and Trimmomatic (v0.38) with parameters (ILLUMINACLIP:TruSeq3-SE.fa:2:15:10 LEADING:30 TRAILING:30 MINLEN:30) to trim any remaining adapters or bases with low quality scores and remove reads shorter than 30 nt. Read alignment and gene counting were performed with STAR (v2.7.0f) [12] using the Rattus norvegicus Rnor 6.0 genome and Annotation 106 from NCBI. Read counts per gene were generated using the featureCounts [6] function from subread v1.6.3 with default parameters except -s 2 to indicate reverse-stranded reads. Of the ~ 97 million raw reads yielded per sample, over 92% of them uniquely aligned to the Rnor 6.0 genome. The proportion of reads in genes was 67.5 - 69% and the extremely high sequencing depth resulted in an average of 62.58 - 70.76 million reads per sample (Fig. S5). We used R (v3.6.2) (R Core Team, 2019-12-12) for all statistical analyses. The TMM (trimmed mean of M-values) normalization method [7] in the edgeR package [8] was used to normalize reads to account for differences arising from total number of reads and RNA composition differences. NCBI Rnor 6.0 Annotation 106 gene models have a total of 38,445 genes, and 22,372 genes were filtered out for no or low expression (0.5 counts per million in at least 3 samples), leaving 16,073 genes for differential expression analysis. We estimated sample-specific quality weights [13] and observational (gene)-level weights using the voomWithQualityWeights function in the limma package [14], which also transformed the counts appropriately for linear modeling [15]. The transformed values and weights were tested for differential expression using an empirical Bayes method, false discovery rates were evaluated, and FDR < 0.2 was chosen for pathway analysis. The list of genes satisfying the above criteria were fed into Ingenuity Pathway Analysis software (IPA®, QIAGEN Bioinformatics, Redwood City, CA), and the resulting top networks (Fig. S6) were analyzed for candidate genes that were then validated using qPCR to confirm RNA-seq results. We validated the RNA-seq data individually in the same animals where sequencing was done. In addition, several genes were also validated in a separate cohort of animals. Therefore, the number of animals is different in Fig. 2B.

Supplementary Results:

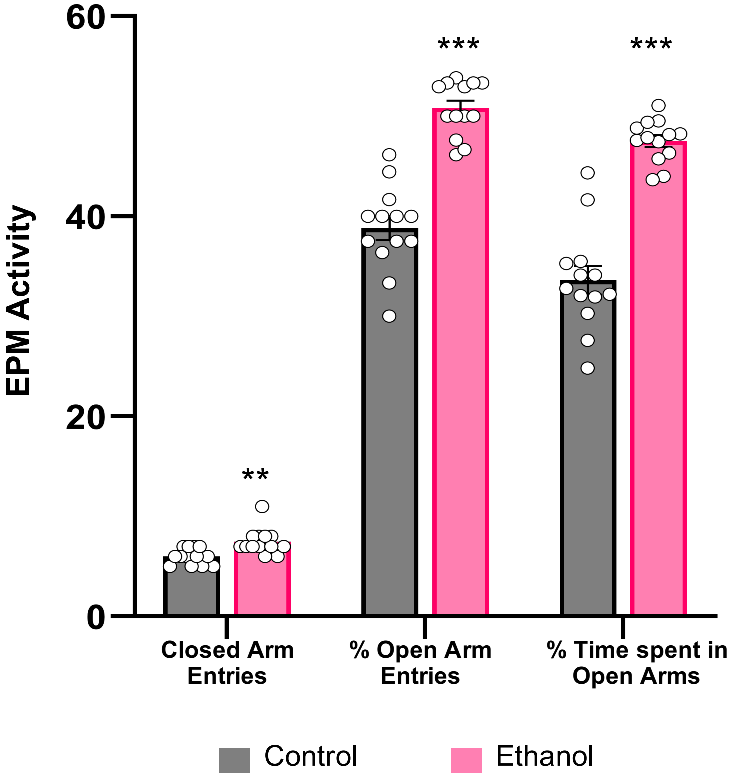

Fig. S1. Effect of acute ethanol exposure on anxiety-like behaviors in adult male rats. This behavior was measured 1hr after acute ethanol (1 g/kg: IP) exposure using Elevated Plus Maze (EPM) exploration test. Values are the mean ± SEM. (n=13; two tailed t-test; **p <0.01; ***p < 0.001).

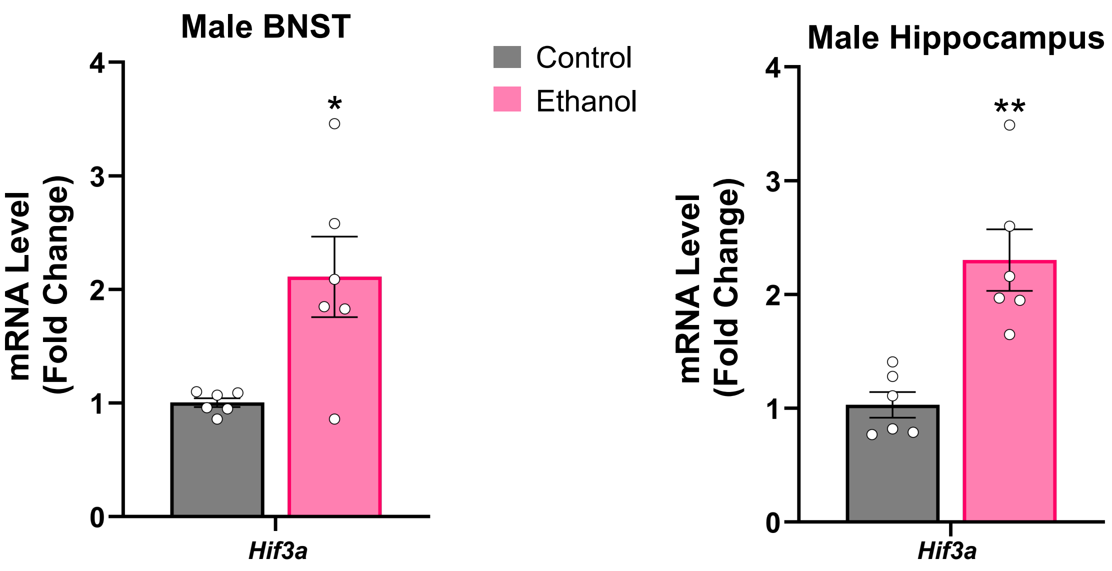

Fig. S2. Effect of acute ethanol exposure on mRNA levels of *Hif3a* in the bed nucleus of the stria terminalis (BNST) and hippocampus of adult male rats. Values are the mean ± SEM. (n=6; two tailed t-test; *p <0.05; **p < 0.01).

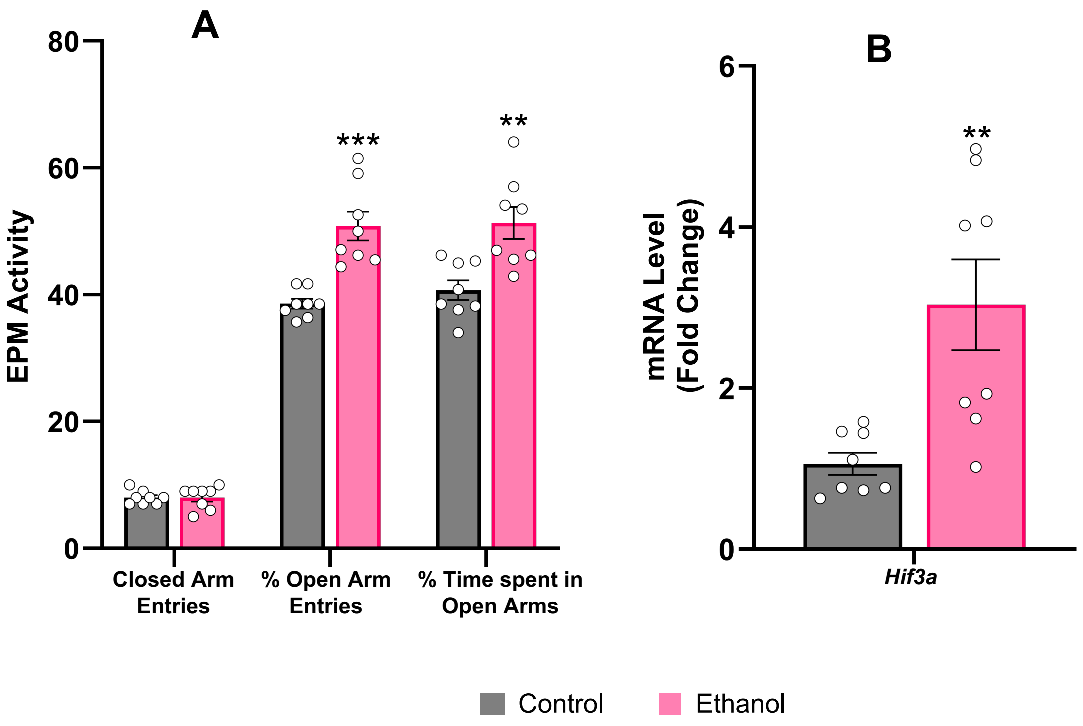

Fig. S3. Effect of acute ethanol exposure on anxiety-like behaviors (**A**) and mRNA levels of *Hif3a* in the amygdala (**B**) of adult female rats. Anxiety behavior was measured 1hr after acute ethanol (1 g/kg: IP) exposure using Elevated Plus Maze (EPM) exploration test. Values are the mean ± SEM. (n=8; two tailed t-test; **p <0.01; ***p < 0.001).

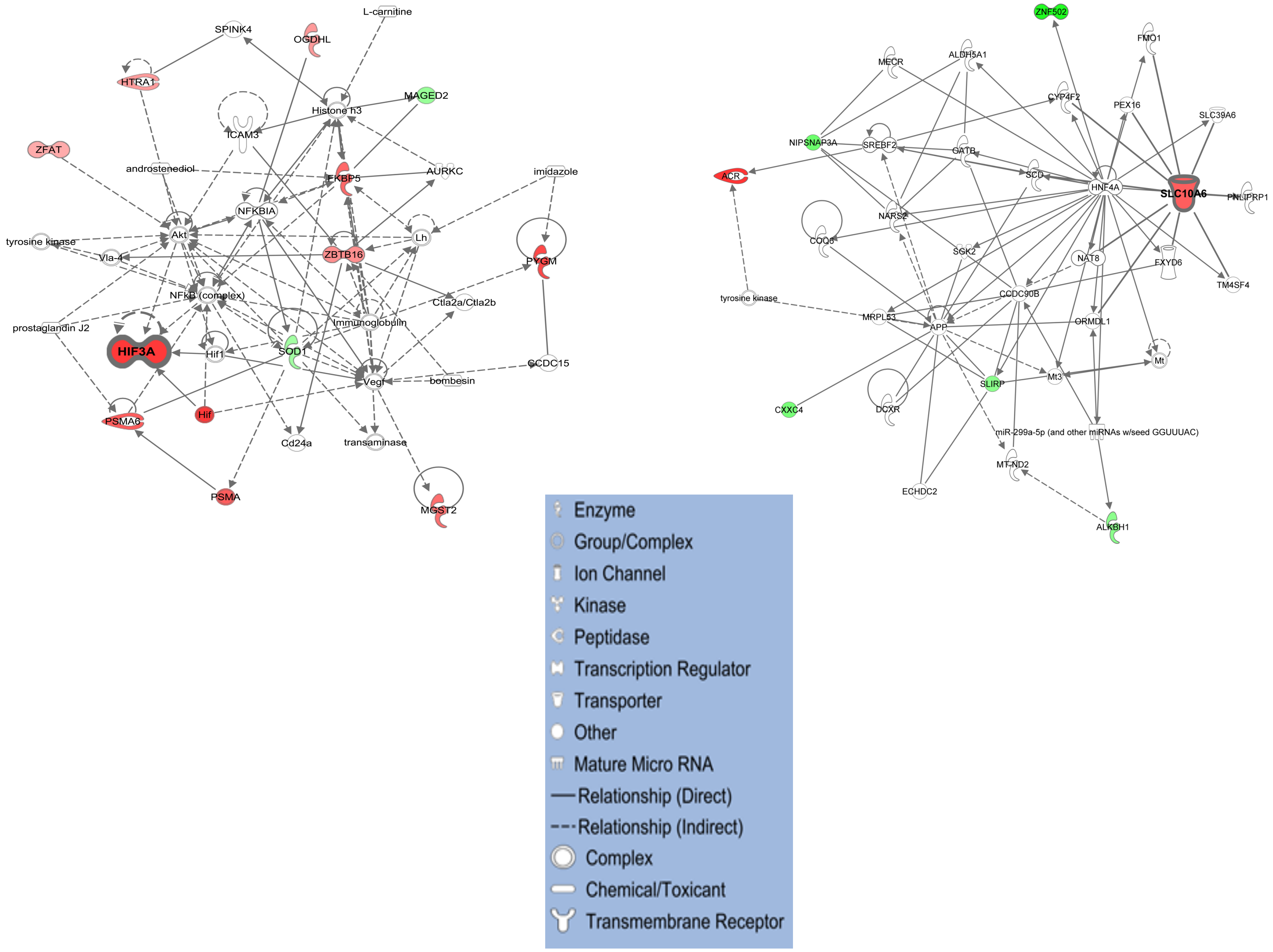

Fig. S4. Ingenuity pathway analysis (IPA) was performed on the list of differentially altered ATAC-seq peaks associated with promoter of the genes (FDR < 0.2) in the amygdala, resulting in two networks, one containing *Hif3a* and other containing *Slc10a6*. The colors indicate increased ATAC peaks (RED) versus decreased ATAC peaks (GREEN) of the epigenome in ethanol-treated compared to control rats. The shapes denote specific protein subtypes as indicated in the inset box legends.

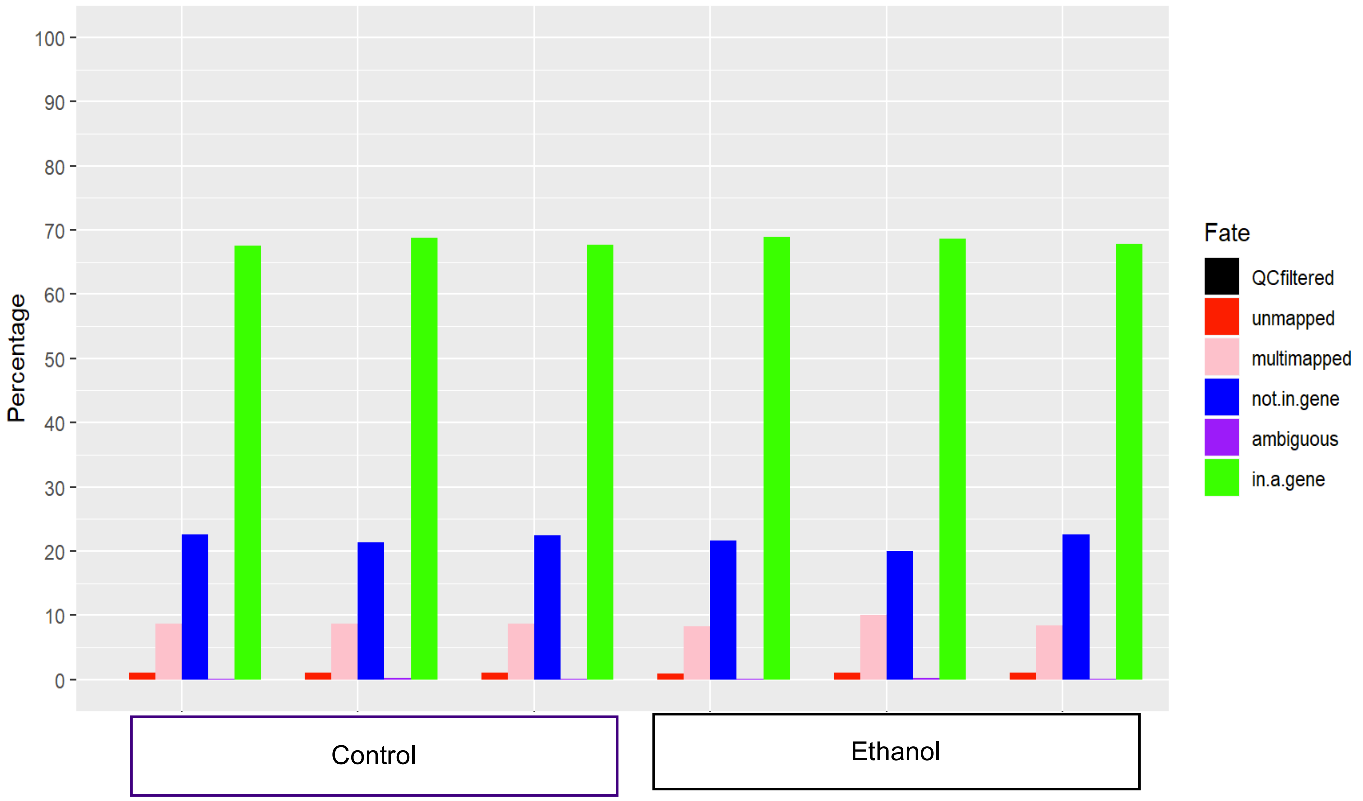

Fig. S5. RNA-seq data analysis in the amygdala of acute ethanol treated rats. Breakdown of the proportion of total reads in each fate for each sample from the RNA-seq analysis is shown. The 6 different read fates (as indicated in the key) are as follows: 1. Filtered out after QC trimming if < 30 bp; 2. Did not align to the genome; 3. Did align, but in more than one location (not "unique"); 4. Did align uniquely, but not within a known gene; 5. Did align uniquely, but to a region covered by two genes ("ambiguous"); 6. Aligned uniquely within one gene and counted as a read for that gene. Overall, all 6 samples have consistent proportions of reads with ~0.68 present in genes.

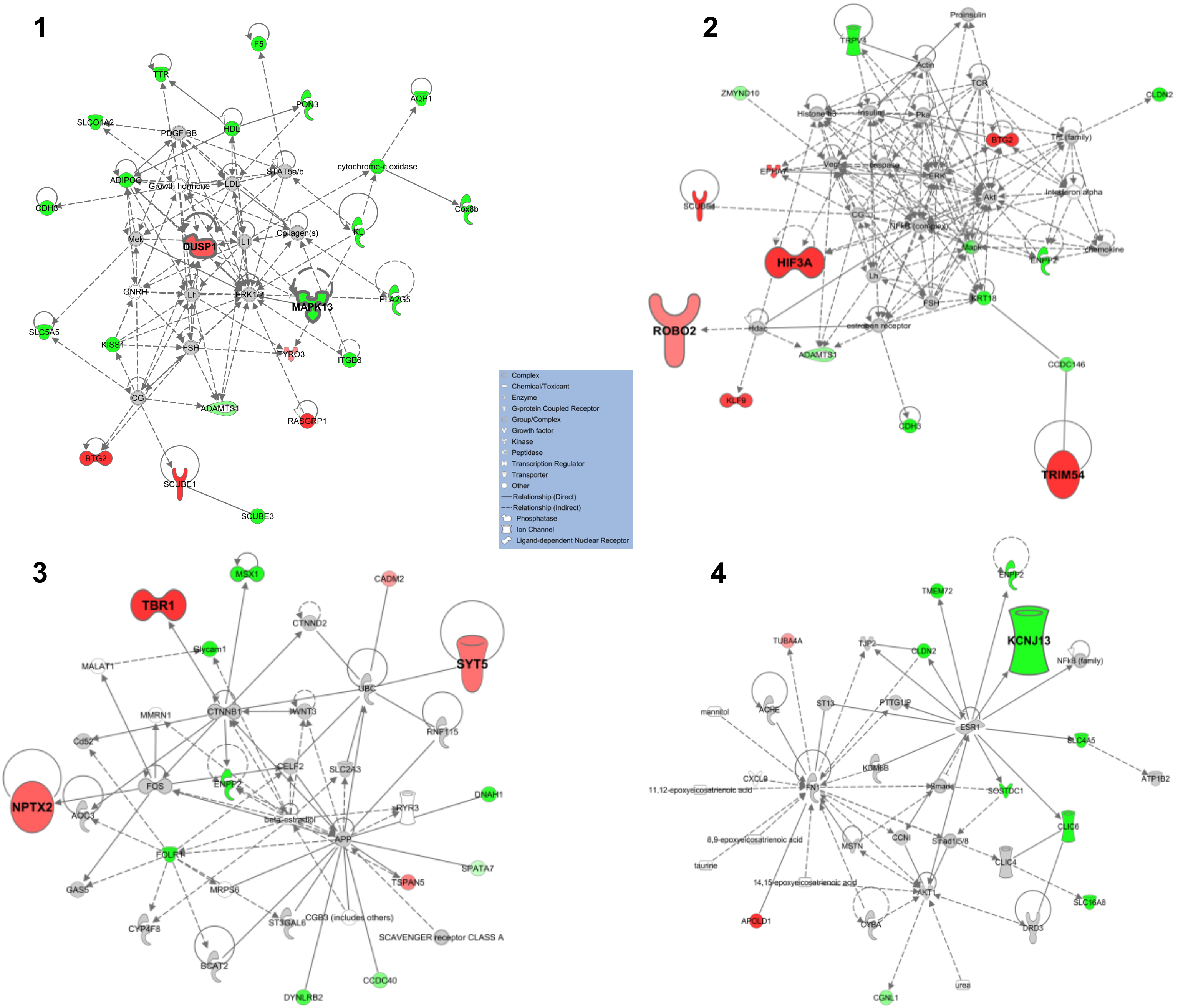

Fig. S6. Ingenuity pathway analysis (IPA) was performed on the list of differentially expressed transcripts (FDR < 0.2) in the amygdala, and the top 4 networks are shown here (1-4). The colors indicate increased expression (RED) versus decreased expression (GREEN) of the transcripts in ethanol-treated compared to control within the RNA-seq dataset. The shapes denote specific protein subtypes as indicated in the inset box legends.

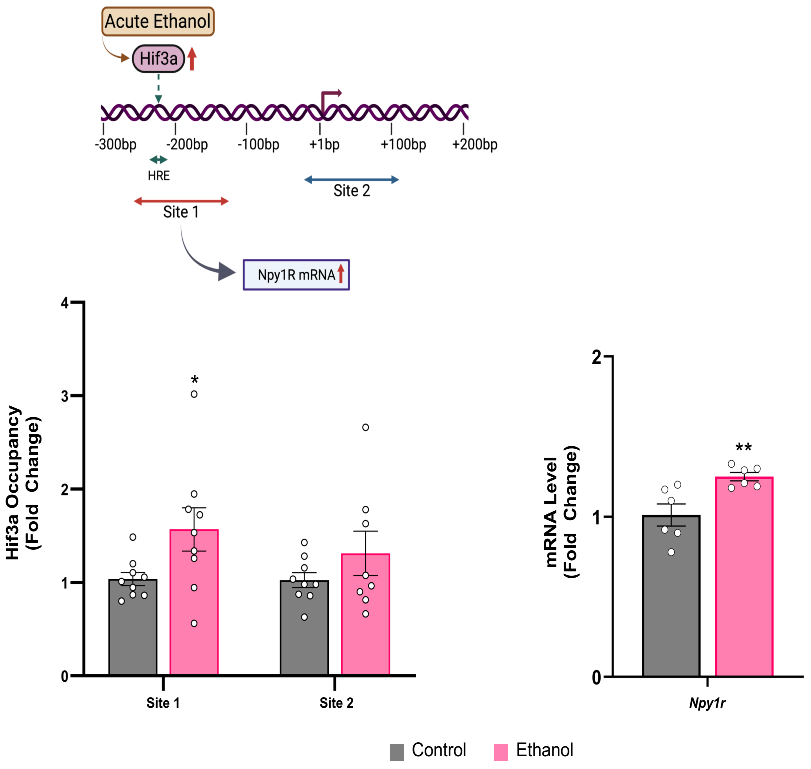

Fig. S7. Representation of neuropeptide Y1 receptor (*Npy1r*) gene transcriptional control region showing the sites where HIF3A protein occupancy was measured (**A**). Effect of acute ethanol exposure on occupancy of HIF3A protein at *Npy1r* gene promoter sites (**B**) and mRNA levels (**C**) of *Npy1r* in the amygdala of adult male rats. Values are the mean ± SEM. (n=6-9; two tailed t-test; *$p < 0.05$; **$p < 0.01$). HRE, Hypoxia response element.

Supplementary Tables:

Table S1. ATAC-seq fold changes of all peaks in the amygdala (Acute ethanol vs Control). Table shows fold changes along with the p-values (please see attached xlsx file for table S1).

Table S2. Footprinting analysis of ATAC peak motifs in the amygdala.
Footprinting data for 41 motifs derived from FIMO analysis of 572 vertebrate motifs from the JASPAR database. The 41 motifs satisfied the following criteria, q<0.01 and >50% enrichment (log2 ratio > 0.585). Table shows the motif consensus sequence ID's along with the p-values.

Motif ID	Motif Name	Total motif hits	Unhit regions	Hits in diff_peaks.list	Unhit in diff_peaks.list	Enrichment (log2 ratio)	P-value	q-value
MA0113.3	NR3C1	25426	93410	165	180	1.160465192	1.878E-27	1.074E-24
MA0007.3	Ar	29198	89638	171	174	1.012430457	1.517E-23	4.339E-21
MA0727.1	NR3C2	25635	93201	156	189	1.067734815	1.553E-22	2.961E-20
MA0490.1	JUNB	30826	88010	147	198	0.715972142	1.528E-11	2.185E-09
MA0489.1	JUN(var.2)	31175	87661	144	201	0.669982935	3.265E-10	3.113E-08
MA0845.1	FOXB1	28383	90453	134	211	0.701509598	5.128E-10	3.667E-08
MA1130.1	FOSL2::JUN	27468	91368	131	214	0.716118626	4.732E-10	3.667E-08
MA0683.1	POU4F2	21177	97659	107	238	0.799416253	2.047E-09	1.032E-07
MA1137.1	FOSL1::JUNB	27476	91360	128	217	0.682275504	3.98E-09	1.751E-07
MA1141.1	FOS::JUND	30004	88832	136	209	0.642755487	5.541E-09	2.264E-07
MA1142.1	FOSL1::JUND	27016	91820	125	220	0.672417694	1.059E-08	4.038E-07
MA0790.1	POU4F1	21507	97329	105	240	0.749886713	2.005E-08	6.746E-07
MA0791.1	POU4F3	21073	97763	103	242	0.751552307	2.766E-08	0.000000879
MA0032.2	FOXC1	30622	88214	135	210	0.602694579	4.046E-08	0.000001218
MA1124.1	ZNF24	30979	87857	136	209	0.59661976	4.516E-08	0.000001228
MA1128.1	FOSL1::JUN	29930	88906	132	213	0.603249334	6.355E-08	0.000001515
MA0476.1	FOS	30431	88405	133	212	0.590188186	9.414E-08	0.000002154
MA0684.1	RUNX3	26117	92719	118	227	0.638101355	1.382E-07	0.000002726
MA0868.1	SOX8	26093	92743	117	228	0.627149388	2.454E-07	0.000004387
MA0099.3	FOS::JUN	27449	91387	121	224	0.60255714	3.445E-07	0.000005796
MA1150.1	RORB	26720	92116	118	227	0.605170535	4.857E-07	0.000007509
MA0614.1	Foxj2	23284	95552	105	240	0.635354088	0.000001113	0.00001592
MA0913.1	Hoxd9	24590	94246	109	236	0.610560083	0.000001437	0.00001912
MA0070.1	PBX1	19121	99715	90	255	0.697142614	0.000001574	0.00002001
MA1143.1	FOSL1::JUND(var.2)	14059	104777	71	274	0.798700632	0.000002571	0.00002896
MA0132.2	PDX1	12364	106472	64	281	0.834301934	0.000004107	0.00004121
MA0722.1	VAX1	13164	105672	67	278	0.809938752	0.000004086	0.00004121
MA0723.1	VAX2	13099	105737	66	279	0.795384935	0.00000683	0.00006301
MA0611.1	Dux	20744	98092	93	252	0.626912193	0.000007831	0.0000711
MA0902.1	HOXB2	14272	104564	70	275	0.75654299	0.000008405	0.00007457
MA0705.1	Lhx8	13514	105322	66	279	0.750386853	0.0000185	0.0001348
MA0756.1	ONECUT2	12981	105855	64	281	0.764045967	0.00001909	0.0001348
MA0679.1	ONECUT1	10871	107965	55	290	0.80132255	0.0000413	0.0002544
MA0838.1	CEBPG	6726	112110	37	308	0.922080175	0.0001751	0.0008634
MA0886.1	EMX2	10591	108245	50	295	0.701464868	0.0005028	0.00204
MA0700.1	LHX2	14042	104794	62	283	0.604895371	0.0006024	0.00236
MA0725.1	VSX1	13739	105097	60	285	0.589061088	0.0009863	0.003482
MA0726.1	VSX2	13739	105097	60	285	0.589061088	0.0009863	0.003482
MA0837.1	CEBPE	6936	111900	34	311	0.755734531	0.002404	0.007023
MA0680.1	PAX7	8316	110520	39	306	0.691888049	0.0026	0.007399
MA0628.1	POU6F1	8466	110370	39	306	0.666097319	0.003694	0.009874

Table S3. RNA-seq fold changes in the amygdala (Acute ethanol vs Control) (please see attached xlsx file for Table S3).

Table S4. Primers used in this study are included in this table.

**RNA ANALYSIS**	
Hif3a mRNA_F	CATCTGCGTCCACTTCCTGA
Hif3a mRNA_R	GGGTCTGCGAGTATGTTGCT
Sgk1 mRNA_F	CAGAAGGACCGGACCAAGAG
Sgk1 mRNA_R	ACCGGCTCCTCAGTAAACTC
Syt5 mRNA_F	TCCCCACATGACTCTCCCTA
Syt5 mRNA_R	TTCCCAACTTCTCCACCTCC
Nptx2 mRNA_F	CAAGGCATCTATTCCCGAGTT
Nptx2 mRNA_R	CCACCAAAGAACAAAGCAGTAAG
Tbr1 mRNA_F	GGAAGTGAATGAGGACGGCA
Tbr1 mRNA_R	GCCCGTGTAGATCGTGTCAT
Robo2 mRNA_F	CCAGCTCTCACAACAGCTCA
Robo2 mRNA_R	AGGAGGAGGAGGTAGAGGGA
Slc10a6 mRNA_F	TTAAACTTCGCCCTGGCCTA
Slc10a6 mRNA_R	AGAGCTGATGTCTTGGCACTC
Trim54 mRNA_F	GAAATGTTGCGGACCATCGA
Trim54 mRNA_R	GAGTCGGATCAGTGCAAACC
Dusp1 mRNA_F	CTCCCGAGTTCCACTGAGTT
Dusp1 mRNA_R	AGAGTCCTTTCCCTTCTGCC
Kcnj13 mRNA_F	TTTCGTTGTCCACTGGCTTG
Kcnj13 mRNA_R	AGAACGCAGCTGTGAAACTG
Mapk13 mRNA_F	GCGGAGATGACTGGCTATG
Mapk13 mRNA_R	TCCACCGTCTGGTTGTAATG
P2rx6 mRNA_F	TGGACACGAAAGGCTCTGAC
P2rx6 mRNA_R	CTGCCTGCCCAGTGACAAG
Npy1r mRNA_F	GGCGAACAGACGGATTCTTTA
Npy1r mRNA_R	CAACCCTGGAGAACAGAGTTGA
Hprt1 mRNA_F	TCCTCAGACCGCTTTTCCCGC
Hprt1 mRNA_R	TCATCATCACTAATCACGACGCTGG
**CHIP/DNA METHYLATION**	
Hif3a_F	CTCTCCGTGCTCCATCTCTC
Hif3a_R	CTTCTCCCGGACTTAGCTGT
Slc10a6_F	CTCAGACTCAGCTCTCCAGG
Slc10a6_R	TCCCTCCCAACACAATTCGA
Npy1r Site1_F	CATCCAGGATCATGTGACGG
Npy1r Site1_R	TCTGACAGTGTGGCTTACGT
Npy1r Site2_F	TCAGACACCCGCATCTTTCT
Npy1r Site2_R	TTCAACACAGGCGAACAGAC

Supplementary References:

1. Li H. Aligning sequence reads, clone sequences and assembly contigs with BWA-MEM. *arXiv preprint arXiv* 2013;**1303**:3997.
2. Buenrostro JD, Giresi PG, Zaba LC, Chang HY, Greenleaf WJ. Transposition of native chromatin for fast and sensitive epigenomic profiling of open chromatin, DNA-binding proteins and nucleosome position. *Nat Methods* 2013;**10**:1213-1218.
3. Wysoker A, Tibbetts K, Fennel T. 2013. Picard tools version 1.90 http://picard.sourceforge.net.
4. Zhang Y, Liu T, Meyer CA, Eeckhoute J, Johnson DS, Bernstein BE et al. Model-based analysis of ChIP-Seq (MACS). *Genome Biol* 2008; **9**: R137.
5. Quinlan, AR, Hall IM. BEDTools: a flexible suite of utilities for comparing genomic features. *Bioinformatics* 2010;**26**:841-842.
6. Liao Y, Smyth GK, W Shi W. featureCounts: an efficient general purpose program for assigning sequence reads to genomic features. *Bioinformatics* 2014;**30**:923-930.
7. Robinson MD, Oshlack A. A scaling normalization method for differential expression analysis of RNA-seq data. *Genome Biol* 2010;**11**:R25.
8. Robinson MD, McCarthy DJ, Smyth GK. edgeR: a Bioconductor package for differential expression analysis of digital gene expression data. *Bioinformatics* 2010;**26**:139-140.
9. Li H, Handsaker B, Wysoker A, Fennell T, Ruan J, Homer N et al. The Sequence alignment/map format and SAMtools. *Bioinformatics* 2009;**25**:2078-2079.
10. Grant CE, Bailey TL, Noble WS. FIMO: scanning for occurrences of a given motif. *Bioinformatics* 2011;**27**:1017-1018.
11. Khan A, Fornes O, Stigliani A, Gheorghe M, Castro-Mondragon JA, van der Lee R et al. JASPAR 2018: update of the open-access database of transcription factor binding profiles and its web framework. *Nucleic Acids Res* 2018;**46**(D1):D260-D266.
12. Dobin A, Davis CA, Schlesinger F, Drenkow J, Zaleski C, Jha S et al. STAR: ultrafast universal RNA-seq aligner. *Bioinformatics* 2013;**29**:15-21.
13. Liu R, Holik AZ, Su S, Jansz N, Chen K, Leong HS et al. Why weight? modelling sample and observational level variability improves power in RNA-seq analyses. *Nucleic Acids Res* 2015;**43**:e97.
14. Law CW, Chen Y, Shi W, Smyth GK. voom: Precision weights unlock linear model analysis tools for RNA-seq read counts. *Genome Biol* 2014;**15**:R29.
15. Ritchie ME, Phipson B, Wu D, Hu Y, Law CW, W Shi et al. Limma powers differential expression analyses for RNA-sequencing and microarray studies. *Nucleic Acids Res* 2015;**43**:e47.
